# Early Detection of Neurodevelopmental Disorders of Toddlers and Postnatal Depression by Mobile Health App: Observational Cross-sectional Study

**DOI:** 10.2196/38181

**Published:** 2022-05-16

**Authors:** Fabrice Denis, Laura Maurier, Kevin Carillo, Roxana Ologeanu-Taddei, Anne-Lise Septans, Agnes Gepner, Florian Le Goff, Madhu Desbois, Baptiste Demurger, Denise Silber, Jean-David Zeitoun, Guedalia Peretz Assuied, Olivier Bonnot

**Affiliations:** 1 Institut Inter-Regional Jean Bernard ELSAN Le Mans France; 2 Toulouse Business School Toulouse France; 3 Kelindi Lille France; 4 Basil Strategies Paris France; 5 VRforHealth Paris France; 6 Centre d’Epidémiologie Clinique Hôtel Dieu Hospital Assistance Publique-Hopitaux de Paris Paris France; 7 Service de Pédopsychiatrie Centre Hospitalier des Pyrénées Pau France; 8 Service de Pédopsychiatrie Centre Hospitalier Universitaire de Nantes Nantes France

**Keywords:** early detection, NDD, neurodevelopmental disorder, ASD, autism spectrum disorder, PND, postnatal depression, mHealth, mobile health, real-world study, autism, parenting, pediatric, children, mobile phone, smartphone, mobile health app, digital health intervention, digital product, algorithm

## Abstract

**Background:**

Delays in the diagnosis of neurodevelopmental disorders (NDDs) in toddlers and postnatal depression (PND) in mothers are major public health issues. In both cases, early intervention is crucial.

**Objective:**

We aimed to assess if a mobile app named Malo can reduce delay in the recognition of NDD and PND.

**Methods:**

We performed an observational, cross-sectional, data-based study in a population of young parents with a minimum of 1 child under 3 years of age at the time of inclusion and using Malo on a regular basis. We included the first 4000 users matching the criteria and agreeing to participate between November 11, 2021, and January 14, 2022. Parents received monthly questionnaires via the app, assessing skills on sociability, hearing, vision, motricity, language of their infants, and possible autism spectrum disorder. Mothers were also requested to answer regular questionnaires regarding PND, from 4-28 weeks after childbirth. When any patient-reported outcomes matched predefined criteria, an in-app notification was sent to the user, recommending the booking of an appointment with their family physician or pediatrician. The main outcomes were the median age of the infant at the time of notification for possible NDD and the median time of PND notifications after childbirth. One secondary outcome was the relevance of the NDD notification for a consultation as assessed by the physicians.

**Results:**

Among 4242 children assessed by 5309 questionnaires, 613 (14.5%) had at least 1 disorder requiring a consultation. The median age of notification for possible autism spectrum, vision, audition, socialization, language, or motor disorders was 11, 9, 17, 12, 22, and 4 months, respectively. The sensitivity of the alert notifications of suspected NDDs as assessed by the physicians was 100%, and the specificity was 73.5%. Among 907 mothers who completed a PND questionnaire, highly probable PND was detected in 151 (16.6%) mothers, and the median time of detection was 8-12 weeks.

**Conclusions:**

The algorithm-based alert suggesting NDD was highly sensitive with good specificity as assessed by real-life practitioners. The app was also efficient in the early detection of PND. Our results suggest that the regular use of this multidomain familial smartphone app would permit the early detection of NDD and PND.

**Trial Registration:**

ClinicalTrials.gov NCT04958174; https://clinicaltrials.gov/ct2/show/NCT04958174

## Introduction

Electronic patient-reported outcomes (ePROs) by smartphone apps have demonstrated their value in the early detection of disease and relapse as well as for prevention or triage of patients in several diseases [1-3]. Patients report symptoms using a dedicated questionnaire. When some criteria meet a pre-established threshold, the prescribing professional is notified and can intervene. Thus, ePROs can reduce delays in diagnosis and treatment while alleviating the burden of monitoring.

After birth, the mother-child dyad can be impacted by impairments that are either undetected or detected too late. Among these impairments, a neurodevelopmental disorder (NDD) such as autism spectrum disorder (ASD) affects 1 in 166 children [4]. The average time to diagnosis is approximately 4 years, whereas consensus statements indicate that a diagnosis could be made as early as 12 or 18 months of age [5-9]. Interestingly, parents are the main contributors to the NDD screening of their children [10]. Other disorders that deserve early screening are hearing disorders, which are observed in 1 in 300 children at age 3 years, and the main visual disorder in toddlers, amblyopia, which is observed with a prevalence of 3% [11-14]. It is, therefore, crucial to provide parents with screening tools and to recommend that they consult a physician at the first symptoms.

Postnatal depression (PND) of mothers is another good example of an underdiagnosed disorder with severe consequences. PND—an episode of depression occurring during the first year after childbirth—has a prevalence of 17.7% and may have a negative impact on the synchrony or receptivity loop that is crucial to the proper neurodevelopment of the baby [15,16].

All these disorders can benefit tremendously from early detection by ePRO questionnaires for parents and their children, which would enable early intervention.

We thus developed Malo, an “all-in-one” multidomain digital health record ePRO app for smartphones, aiming to facilitate early screening of NDDs in children from birth to age 3 years and PND in mothers. We assessed the performance of this app in an observational cross-sectional, data-based study.

## Methods

### Ethics Approval

We ran an ecological, observational, cross-sectional, data-based study. Our study was approved by the French National Health Data Institute (HDH approval number F20210420115840), which ensures ethical conduct in human subject research regarding data confidentiality and safety.

### Population

Our users were recruited during a 2-month period, following a French national media campaign that was disseminated through social media between November 11 and 18, 2021. We selected a wide array of networks, both professional (eg, LinkedIn) and nonprofessional (eg, Facebook, Instagram, TikTok, and Twitter). A national and regional press campaign also relayed the following message (in print, audio, and television): “Use Malo to improve follow-up of neurodevelopment of your toddler and your mental burden.” Finally, we also used Google ads and Facebook ads to encourage recruitment for this study. To participate, individuals were required to download the app (Malo) on the Android or Apple app stores, create an account and electronically confirm their agreement to the applicable terms and conditions of the app, then opt in to our research. Enrollment in the study was strictly optional. Recruitment was open with no exclusion criteria. The only inclusion criteria were to download the app and give informed consent (in-app). The study population target was 4000 users to obtain at least 30 possible cases of ASD screened by the app.

### Data Collection

Data collection was embedded in the app. Data were anonymously collected in a French labelled health data cloud. The approval number for our human subjects review was F20210420115840. Respondents anonymously self-entered the age and gender of their infants. The app also allowed for the entry of the children’s height, weight, vaccination status, medical background, and ongoing or previous treatments. Questionnaires and scales, each containing 25-50 questions assessing neurodevelopment skills, were automatically submitted every month from birth to 9 months, then at 11, 12, 16, 18, 21, 24, 30, and 36 months, and were focused on language, socialization, hearing and vision, and motricity.

Questionnaires and notifications were based on French health authorities’ reports, international recommendations, and experts’ agreements [17,18].

The questionnaire for the screening of postnatal depression was submitted every 21 days between 4 and 28 weeks after childbirth, using a modified questionnaire of the Edinburgh Postnatal Depression Scale adapted to self-assessment.

### Threshold-Based In-App Notification

Notifications were sent automatically to the user if some symptoms matched predefined criteria and a physician consultation was recommended.

Regarding NDDs, once a threshold of concern was reached, 2 types of notifications were sent: type A notifications recommended discussing their symptoms with a general practitioner (GP) and type B notifications recommended contacting a pediatrician.

Regarding maternal depression, there were 3 grades of notifications sent to the mother: grade 0 (score lower than 25) was associated with a message indicating that everything is ok; grade 1 (score between 26 and 50) was associated with a recommendation to talk about symptoms with a close relative; grade 2 (score between 51 and 65) recommended that they quickly discuss their symptoms with a family doctor; and grade 3 (score higher than 65) recommended that they meet a family doctor as soon as possible (Multimedia Appendices 1-4).

The main outcome was the median age of possible NDD notification of infants. The secondary outcomes were (1) the median time of the mothers’ PND notifications after childbirth; (2) the rates of adoption (assessed by the percentage of users who filled in at least 1 questionnaire); (3) user satisfaction regarding app functionality, the relevance of advice received, and the level of support in child follow-up; and (4) the relevance of the NDD notifications assessed by physicians, using a specific optional survey asking parents the following questions:

- In the past month, did your doctor detect a developmental disorder in your child during a follow-up consultation? YES/NO
- If you had a notification by Malo, did you follow the recommendation of the app to visit a physician? YES/NO
- If YES, which health professional did you contact? GP or pediatrician?
- Which of the following reflects the physicians’ reply? (1) The notification is not relevant, (2) the notification is relevant and a medical surveillance of the evolution of the symptom is needed, (3) the advice of an expert is needed, or (4) a treatment is indicated.

### Analysis

The analysis was performed when at least 4000 users downloaded the app and filled in at least 1 infant’s questionnaire of neurodevelopment screening.

Sensitivity, specificity, predictive positive and negative values, and the Youden index of the algorithms triggering notifications of suspected NDDs were calculated according to the physician’s feedback. A notification was considered relevant if a physician suggested a specific medical surveillance of the disorder or the consultation of an expert or a therapist.

Chi-square test was used in 2×2 tables to assess the statistical association between the medical relevance of the notification (relevant or not) and the notification results (notification or no notification of a possible NDD). We also assessed the rate of probable PND of mothers having a score >50 in the survey.

The level of statistical significance was 5% for all statistical tests (exploratory tests).

## Results

### Overview

**Figure 1:**
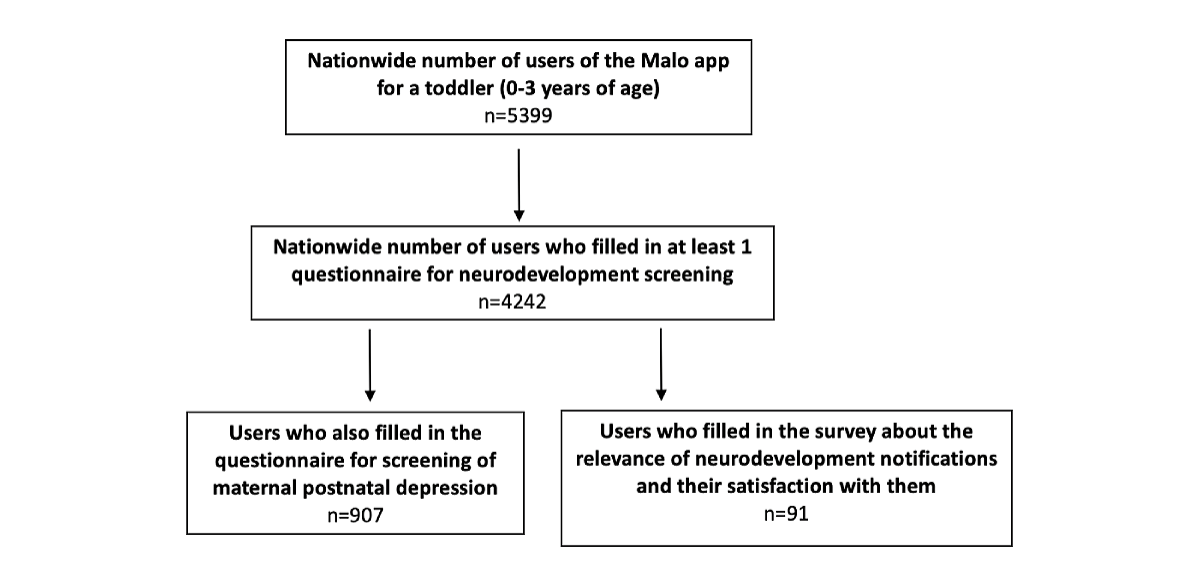
Flowchart of users of the Malo app. Among 91 respondents, 54 received notification of a possible neurodevelopmental disorder requiring a physician visit and reported the physician’s assessment of the relevance of the notification.

Between November 11, 2021, and January 14, 2022, 6426 users downloaded the app, and at least 1 questionnaire was filled in for 4242 children (fill rate=66.0%), leading to the analysis of 5309 questionnaires and 126,539 questions for pediatric neurodevelopment assessment. Data analysis was performed at the end of January 2022 (Figure 1).

The median age of the toddlers assessed by the questionnaires was 3.9 months, and 2202 (51.9%) were boys.

**Figure 2:**
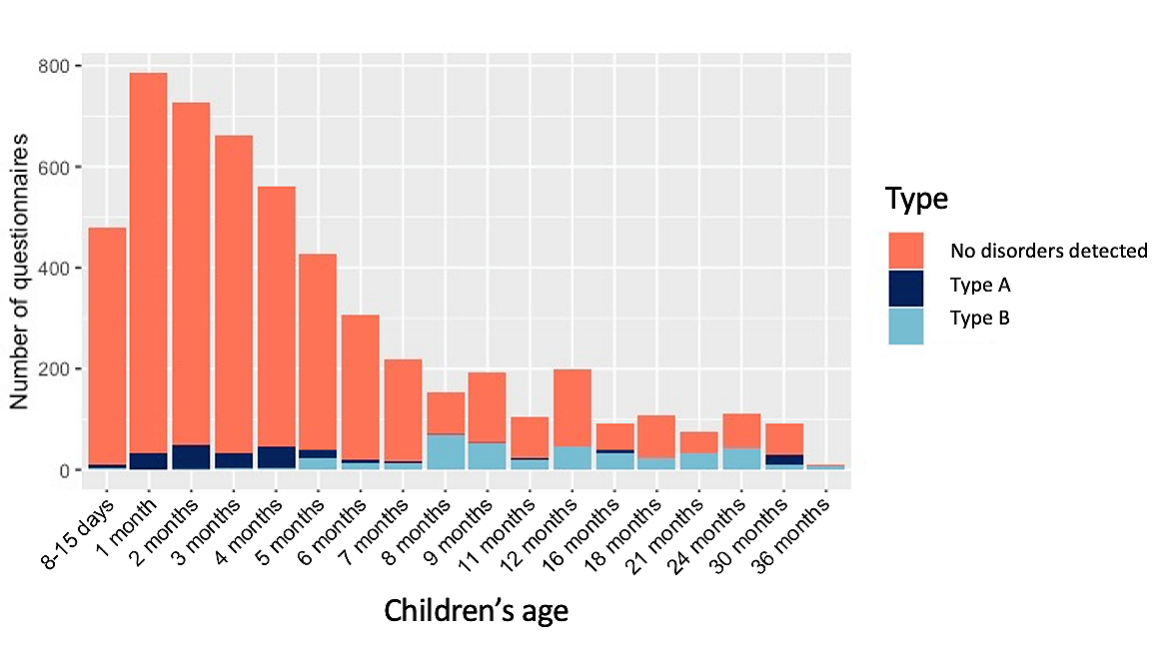
Distribution of the notifications of possible neurodevelopmental disorders and their type according to the toddler’s age. There were 2 types of notifications: type A recommended talking about the symptoms with a general practitioner and type B suggested meeting with a pediatrician.

During the 8 weeks of recruitment, among the 4242 children, 216 (5.1%) had a type A notification of a possible disorder (recommended a GP visit), and 397 (9.4%) had a type B notification (recommended a pediatrician visit) (Figure 2).

There were 0.9% (39/4242) toddlers with notifications for possible ASD, and the median age of alert was 11 months.

**Figure 3:**
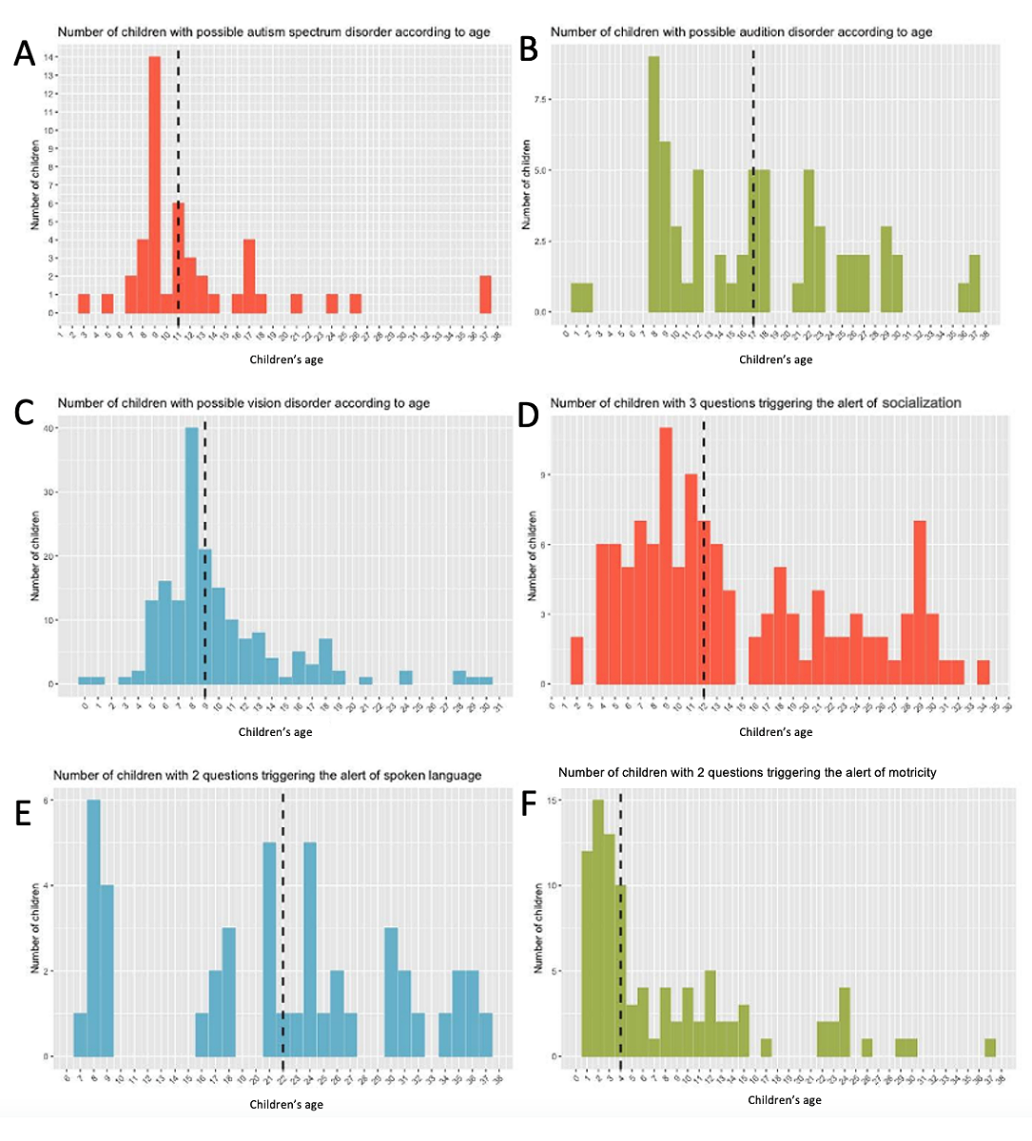
Number of children with possible neurodevelopmental disorders according to age (months): (A) autism spectrum disorder, (B) auditory, (C) visual, (D) socialization, (E) language, and (F) motricity. The dashed line is the median time of detection by the app.

The rates of possible vision and auditory disorders were 11.3% (481/4242) and 1.8% (78/4242), respectively, and the median age of children at the time of such alerts was 9 and 17 months, respectively. The rate of possible socialization disorders was 2.8% (120/4242), and the median age of alerts was 12 months. The rate of possible language disorders was 1.1% (45/4242), and the median age of alerts was 22 months. The rate of possible motricity disorder was 2.2% (95/4242), and the median age of alerts was 4 months (Figure 3).

### Analysis of the Assessment of the Relevance of the Alerts by the Physician

Among the 91 users who agreed to answer the survey concerning the physician consultation, 27 had no alert, and 64 had received an alert of a possible NDD, which suggested a visit to their physician.

Among users who received a notification suggesting a visit to their physician for a neurodevelopmental issue, 84.4% (54/64) answered “YES” to the question “If you had a notification, did you follow the recommendation of the app to visit a physician?” Among users who visited a physician, 51.9% (28/54) met with a family doctor and 48.1 % (26/54) met with a pediatrician (48.1%, 26/54).

The analysis of the clinical relevance of the alerts, as assessed by the physician, showed a sensitivity of 100%, a specificity of 73.5%, a positive predictive value of 70.4%, a negative predictive value of 100%, and a Youden index of 72% (*P*<.001). Among the 38 children with true positive notifications of a possible NDD suggested by the app, medical surveillance of the evolution of the symptoms was proposed in 31 cases (81.6% of relevant notifications), the advice of an expert was needed in 2 cases (5.3%), treatment was immediately initiated in 4 cases (10.5%), and another medical act was executed in 1 case (2.6%).

### Satisfaction Analysis

Among users who filled in the satisfaction survey, 77.4% (82/106) reported that the app improved the follow-up of their child, 95.3% (101/106) found the app easy to use, and 98.1% (104/106) reported that the advice was adapted to the follow-up of the development of their child.

### Screening of PND

**Figure 4:**
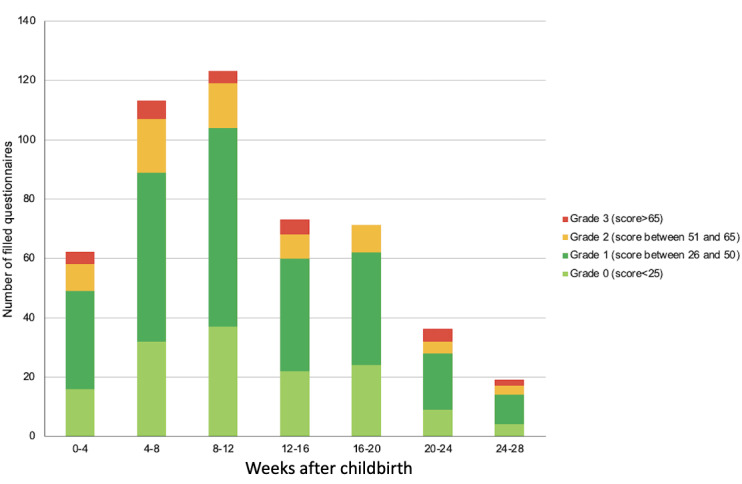
The distribution of the results of the maternal postnatal depression screening according to the number of weeks after childbirth. Only grade 2 or 3 triggered a notification to visit a physician.

Among 907 mothers who completed PND questionnaires, 151 (16.6%) were suspected to have PND. The median time of detection was between 8 and 12 weeks after childbirth, and 370 (40.8%) of the detections occurred before the eighth week after childbirth (Figure 4).

## Discussion

### Principal Findings

Our study is the first to prospectively assess, in a “real-world” manner, the benefit of mother-child dyad follow-up by a dedicated multidomain familial mobile health (mHealth) smartphone app providing early detection of NDDs and maternal PND.

The main result is that 0.9% (39/4242) of toddlers were identified as potentially having ASD, and the median age of the alert was 11 months. This is very close to the 0.6% ASD rate in the general population [4]. Our detection age is at least 3 years earlier than what is usually observed, as the mean age of disease detection is usually late (4-6.8 years for ASD) [10].

EPROs enable users and patients to have relevant clinical effects on many outcomes such as quality of life, early detection of events, and best orientation to specific care even for new diseases such as COVID-19 [1-3,19-21].

In our study, we show that ePROs may help parents to optimize the neurodevelopment follow-up of their children. In a recent survey from France, the identification of the first symptoms of NDDs was done by parents (without a dedicated ePRO) in 61% of cases and by a health professional in only 14% of cases [10]. That is why we have chosen to provide parents with a smartphone app that allows for a relevant and scalable screening of NDDs based on validated questionnaires. The instruments allowed parents to screen for autism spectrum, language, socialization, hearing, vision, and motricity disorders and triggered alerts when the app recommended a consultation. Since early detection of ASD proved to be achievable and stable by 12-18 months of age in a recent study of 1269 infants, we found it worthwhile to provide parents with an instrument for neurodevelopmental skills assessment as early as possible [5-9]. The median ages of notification for possible autism spectrum, vision, and audition disorders were 11, 6, and 18 months, respectively. The median ages of notification for possible socialization, language, and motor disorders were 12, 22, and 4 months, respectively. These results are very encouraging and confirm the feasibility and relevance of familial multidomain screening of NDDs via a smartphone app. Early screening allows for early diagnosis and interventions as reported by works on the efficacy of early treatments of cases among very young children and recent promising studies on early interventions [22-24]. Moreover, the early detection of visual, audition, language, and motor skill disorders is also associated with better prognosis, especially when they are diagnosed before 3 years of age [25-27].

We also performed an analysis of physician feedback after an alert about a possible NDD. Most users (54/64, 84.4%) followed the recommendation of the app to visit their family doctor or pediatrician after an alert. The physician agreed with the relevance of the alert in 70.4% of cases (predictive positive value). Among this 70.4%, the physician triggered a specific medical surveillance in 81.6% of notifications or initiated a treatment or referred parents to an expert. The sensitivity and the negative predictive value of notifications were 100%, and the specificity was 73.5%. Although these data are declarative by users and ASD diagnosis was not directly confirmed by physicians, we can suppose that the specificity of the ASD notifications is close to Pierce et al’s [9] results, showing an overall stability or specificity of an autism spectrum diagnosis of 84% at earlier than 18 months of age through a universal screening program in primary care. In a recent diagnostic accuracy study including 13,511 children aged 11-42 months, Barbaro et al [28] showed an 83% positive predictive value and 99% estimated negative predictive value of the Social Attention and Communication Surveillance-Revised tool for autism identification when it was used by nurses for 12-month-old children. Our results seem to be similar when parents perform a screening using our app.

To improve the neurodevelopment of the child, we added an early PND screening tool to the ePRO instrument because PND is well known to disrupt the crucial mother-infant relationship on which optimal child development depends. It is the most common complication associated with childbirth, and it may exert harmful effects on children such as increased risk of ASD [29]. It is usually underdetected or detected after many months. The early treatment of PND is effective and does not necessarily require drugs to improve symptoms in the earliest stages [30]. Its prevalence in France is 18% and we found that 151 (16.6%) of users had probable PND in our cohort. Interestingly, 40.8% of the detections occurred before the eighth week after childbirth, which is within the recommended time frame to begin treatment for this underdetected disorder [15,16]. As the app sent notifications to the user recommending a visit to a family doctor if depression was suspected, we think that this early screening may contribute to an improvement in care and may reduce the negative impact of PND on pediatric neurodevelopment.

The rate of users who filled in at least 1 questionnaire regarding toddler neurodevelopment was high (66.0%) and the rate of parents who followed recommendations for an early visit to the physician was 84.4% (54/64). This underscores parents’ interest and confidence in this instrument, as the average response rate reported in the literature for general eHealth apps is 49% [31]. We made the choice to incorporate 2 domains of health assessment in a single smartphone to avoid requiring families to use 2 separate apps.

The levels of satisfaction were also high (between 77.4% and 98.1% according to the assessed domains) and contributed to the high rate of adoption. A high level of satisfaction for eHealth solutions is defined as rates higher than 75% [32,33].

### Study Limitations

Limitations of our study are the following. First, sample selection bias is always possible in the absence of randomization, due to social media recruitment modalities and because using the mobile app requires possession of a smartphone. We could have asked users questions about their educational level, practice classification (rural or urban), technical experience, and marital status, but we designed the app to collect as little personal data as possible. However, the very high rate of smartphone penetration in France (92% in a 2018 survey) among people aged 25-39 years led us to believe that the risk of a selection bias associated with smartphone use was low. Nonetheless, we do note that parents without smartphones cannot benefit from the app [34].

Because the social media recruitment strategy could have selected for more employed people in urban areas, we complemented the recruitment with national and regional press campaigns and the support of health insurance companies who also phoned and sent postal mail to members in the required age group. In France, everyone aged 25-45 years has basic and complementary health insurance; therefore, we think we reached as many potential users as possible, regardless of their socioeconomic status. However, the women who agreed to participate in the PND study may more likely have been first-time mothers with a higher level of education compared to the general population. This was recently observed in a longitudinal study from Italy on predictors of PND [35]. The impact of this potential selection on the PND screening rate is probably low, as the observed incidence result in our study (n=151, 16.6%) is close to the rate in the general population (17.7%) [16]. The second limitation was that the data were declarative by users with a comparative arm, and ASD diagnoses were not directly confirmed by physicians. Third, the attrition rate (ie, the discontinuation of eHealth app use) was not assessed, but it could be interesting to study whether the benefit of early detection of NDD is maintained over time by prolonged use [36].

### Conclusions

To our knowledge, this multidomain mHealth app dedicated to both the early detection of NDDs in toddlers and the early detection of maternal PND is the first app with real-life data of clinical relevance on this topic. Results suggest that a multidomain familial mHealth app is suitable and effective for regular use in the mother-child dyad follow-up.

## Appendix

Multimedia Appendix 1 Screenshot of the Malo app showing questions about motricity.

Multimedia Appendix 2 Screenshot of the Malo app showing that everything is ok.

Multimedia Appendix 3 Screenshot of the Malo app showing questions about postnatal depression.

Multimedia Appendix 4 Screenshot of the Malo app showing notification to visit a physician for potential postnatal depression.

## Abbreviations

ePRO: electronic patient-reported outcome
GP: general practitioner
mHealth: mobile health
NDD: neurodevelopmental disorder
PND: postnatal depression
