# Supplementary figures and images for: Early Detection of Neurodevelopmental Disorders of Toddlers and Postnatal Depression by Mobile Health App: Observational Cross-sectional Study
Source: JMIR Mhealth Uhealth. 2022 May 16;10(5):e38181. doi: 10.2196/38181 (PMC9152715; doi:10.2196/38181)

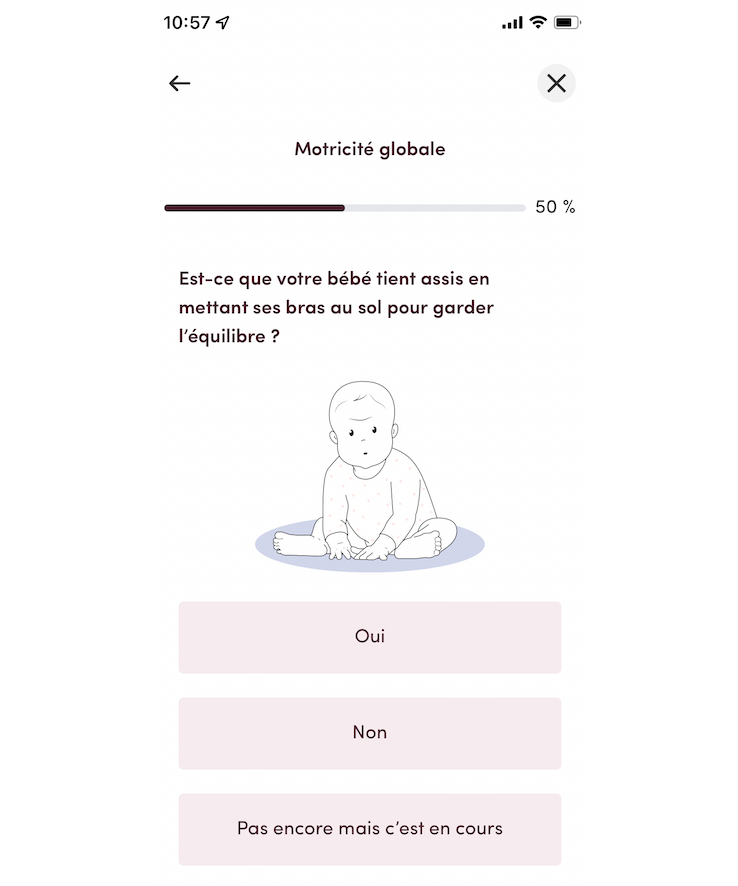

Supplement: Multimedia Appendix 1 [file mhealth_v10i5e38181_app1.png]

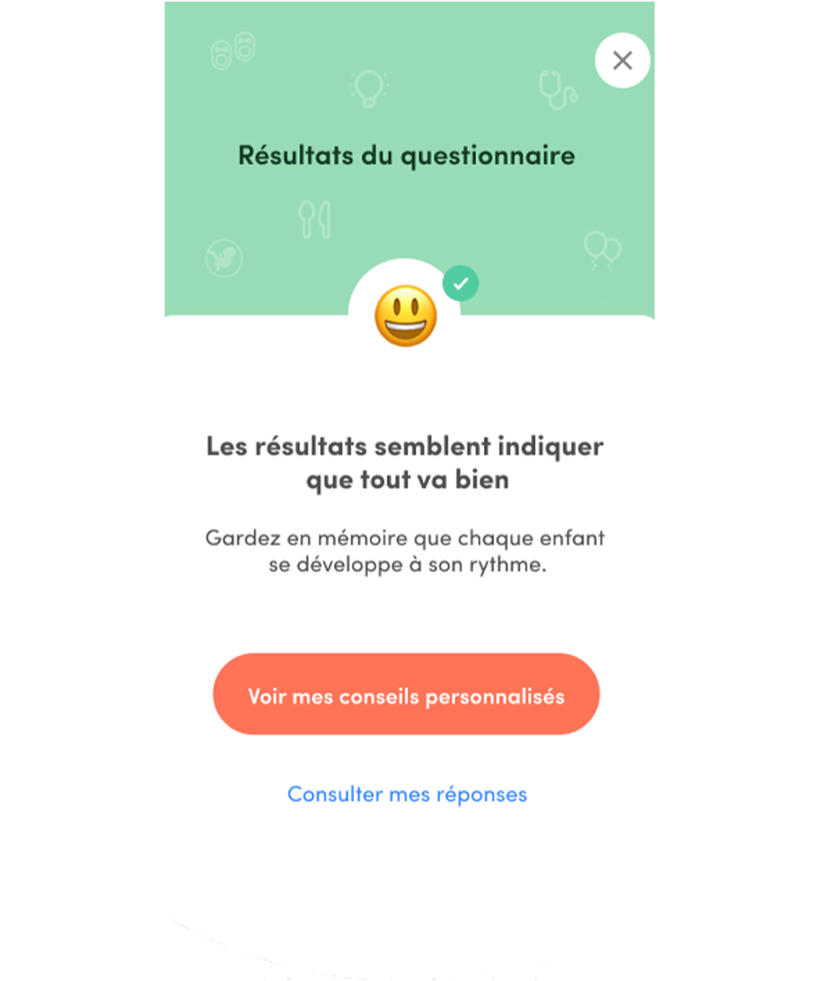

Supplement: Multimedia Appendix 2 [file mhealth_v10i5e38181_app2.png]

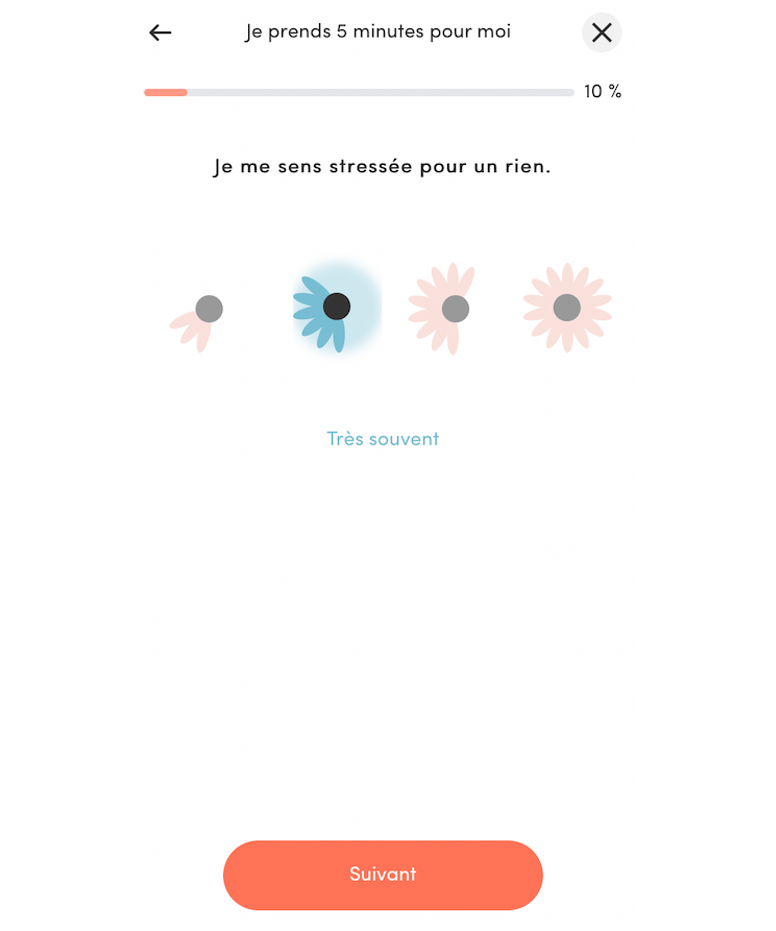

Supplement: Multimedia Appendix 3 [file mhealth_v10i5e38181_app3.png]

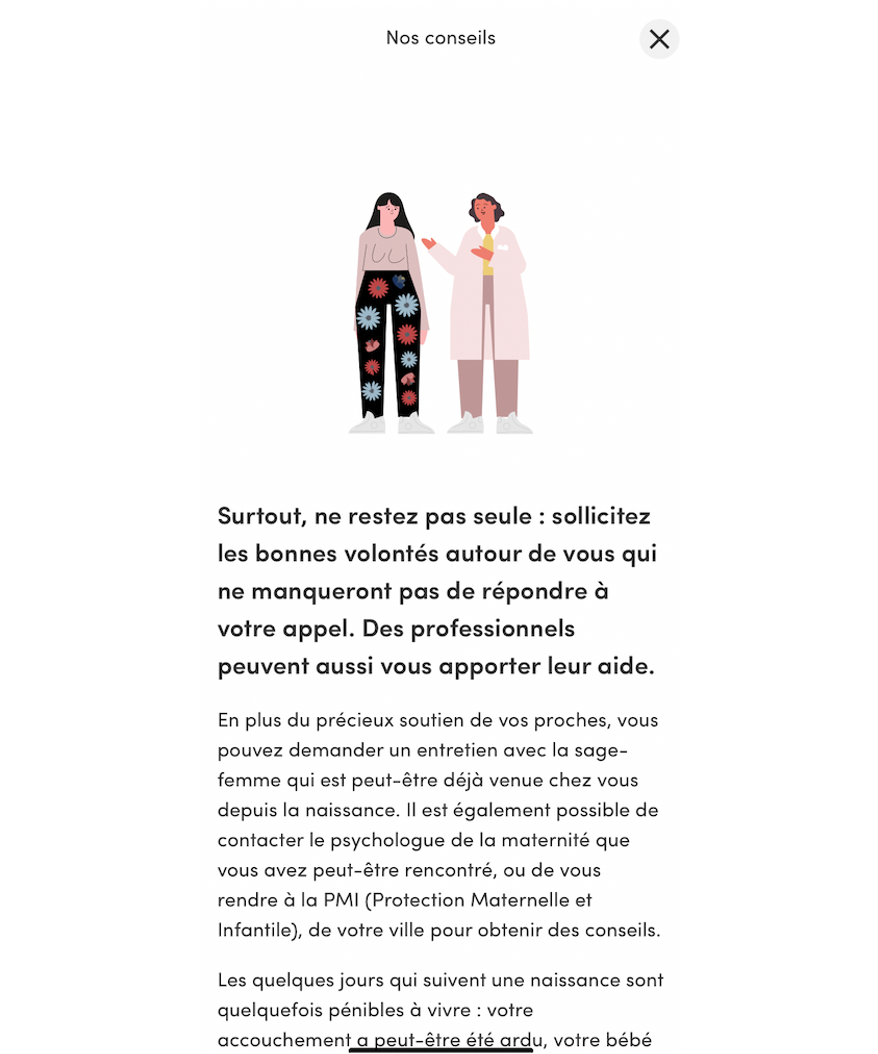

Supplement: Multimedia Appendix 4 [file mhealth_v10i5e38181_app4.png]
